# Generating solutions for charged compact configurations in *f*(*R*) gravity

**DOI:** 10.1371/journal.pone.0349625

**Published:** 2026-07-06

**Authors:** I. Noureen, S. A. Mardan, A. Zahra, I. Anwar

**Affiliations:** 1 Department of Mathematics, Government College Women University Faisalabad, Pakistan; 2 University of Management and Technology, Lahore, Pakistan; 3 Center for Theoretical Physics, Khazar University, Baku, AZ1096, Azerbaijan; 4 IT4Innovations, VSB-Technical University of Ostrava, Ostrava, Czech Republic; Henan Polytechnic University, CHINA

## Abstract

We present a systematic method for generating solutions of charged, spherically symmetric compact objects in modified *f*(*R*) gravity. The stellar interior is modeled with an anisotropic matter distribution, allowing distinct radial and tangential stresses. The modified field equations initially provide three relations among five unknown functions. To construct a consistent system, we impose a linear equation of state together with two geometric constraints: the conformally flat condition (vanishing Weyl tensor) and the Karmarkar condition associated with class-I embedding. These conditions lead to a closed expression for the energy density, which serves as a generating function for the model. Within this framework, the radial pressure, tangential pressure, and anisotropy factor naturally emerge as key drivers in the generation of solutions. The resulting Riccati equation is transformed into a second-order linear homogeneous form, enabling explicit construction of stellar models. Our analysis demonstrates that once a particular solution is identified, entire families of generating solutions can be obtained in *f*(*R*) gravity, with the anisotropy factor playing a central role in their formulation.

## 1 Introduction

Gravity is the fundamental and dominant force that governs the formation, structure, and evolution of stellar bodies. It dictates how stars develop, stabilize, and eventually reach their final stages of evolution. The study of the universe is always an interesting field for researchers and is a highly focused area. A stellar body is in a stable condition when outer forces and the inner-directed gravitational pull of the system are in balance. The existence of neutron stars, black holes, or white dwarfs is the result of the gravitational collapse of compact objects. These stellar remnants are extremely dense in nature [1].

General theory of relativity (GR) introduced by Albert Einstein [2] provides a new conception of the universe. According to Einstein’s theory, it is dynamical system susceptible of mathematical modelling and physical measurements. Before that, it was considered that time and space are the only universal components of Newtonian mechanics. The theory of GR gives coherent and comprehensive information or explanation about space, matter, time, and gravity in weak gravitational fields.

Although GR has been a tremendous success in explaining the gravitational phenomena, several unsolved issues in both contemporary astrophysics and cosmology have led to the exploration of alternative theories of gravity. The late-time accelerated expansion of the universe, which is observable and has not been fully understood, including the dark matter, dark energy, and singularity formation during gravitational collapse, provides evidence that GR needs amendment in the strong-field and large-scale regimes [3,4]. Particularly, gravitational theories beyond GR are best tested by examining compact objects, including neutron stars and other compact stellar forms, in which the high curvature effects are important. These and other arguments have generated the growing interest in higher-order theories of gravity which can include corrections due to higher-order curvature and provide a more comprehensive account of gravitational interactions still consistent with observational constraints [5–7].

The present illustration of the universe indicates some clarification in theoretical work, which fulfills all the requirements and strongly necessitates alternative gravitational theories. Alternate modified theories are based on enlargement and corrections to GR. However, an extended theory of gravity is validated only if it has negligible corrections in weak field regimes. In other words, extended theories of gravity are physically acceptable only if their results reduce to GR in limiting cases [8–11]. A large number of alternative theories of gravity have been proposed till now; they may carry higher order curvature invariants, scalar tensor gravities are also very significant as well as introduction of extra spatial dimensions may be utilized to interpret large scale structures. Such alternative theories include *f*(*R*), *f*(*T*), *f*(*R*,*T*), *f*(*G*,*T*) gravities, Kaluza Klein theory, Brans Dicke theory etc [12–16].

The *f*(*R*) gravity is one of the extended theories, which is an evident and highly motivated generalization of GR: it is achieved by replacing the Ricci scalar *R* in the Einstein Hilbert action by a nonlinear form *f*(*R*). This change adds the higher-order curvature terms, which are important in the strong gravitational fields, e.g., those of compact stellar objects. The structure of *f*(*R*) gravity is especially attractive since it is capable of successfully explaining cosmic acceleration and attempting reasonable corrections to GR without introducing extra exotic matter fields. Moreover, it enables the creation of physically realistic models of stars and makes it easier to study the major characteristics of anisotropy, stability, and energy states. The fact that the appropriate functional forms of *f*(*R*) are flexible enough allows the construction of models that are consistent in theory and have the right observational constraints, thus being a good and solid choice to study the structure and stability of compact objects in modified gravity [17]. Buchdahl [18], who proposed a pioneering extension of Einstein’s theory, investigated nonlinear gravitational Lagrangians in which the Ricci scalar *R* in the Einstein–Hilbert action is replaced by a more general function of curvature. This early work laid the conceptual foundation of modern modified gravity theories and demonstrated that higher-order curvature terms may have important consequences in cosmology and gravitation. In particular, he showed that nonlinear functions of *R* could provide viable alternatives to standard GR in describing large-scale cosmic dynamics.

Many authors have modified Einstein Hilbert (EH) action for this theory by using higher-order curvature invariants [19–21] given as follows:


$$\begin{array}{c} S_{f(R)}=\frac{1}{2\kappa }\int d^{4}x\sqrt{-g}f(R)+S_{M}, \end{array}$$ (1)

where *g* stands for determinant of the metric, κ represents the coupling constant and $S_{M}$ denote the matter field action. Variation of above action through metric variational principle yields covariant form of modified field equations (FEs). A large number of researchers have made great contributions in the study of modified gravity theories, both through the development of analytical and numerical models to investigate the structure and stability of compact objects. Their works form a solid background to explore the impact of features like anisotropy, charge, and curvature corrections on stellar equilibrium in alternative gravity models [22–25].

In relativistic astrophysics the charged gravitating sources is known as important ingredient to study impact of electromagnetic effects that can be induced through Maxwell source. The research of such stellar compact objects, the electromagnetic FEs in static spherically space-times provide the basis for investigation of compact sources comprising heat conduction that covers energy dissipation. Maxwell equations exhibits linearity whereas Einstein equations are highly non-linear differential equations of second order. They are solved for the system with symmetry [26]. For static spherically symmetric interior space-time Solutions of the Einstein-Maxwell FEs is very important to study combined of gravitational pull and electromagnetic forces. It is important to note that compact objects such as neutron stars, strange stars, and other ultra-dense remnants provide one of the best laboratories for testing gravitational theories beyond GR. In these systems, the matter density, spacetime curvature, and internal pressure gradients become extremely large, making curvature corrections predicted by modified gravity potentially significant. Therefore, obtaining interior solutions for charged and anisotropic stellar configurations is not only mathematically useful but also essential for understanding how modified gravity may alter observable quantities such as density, radial and tangential pressure and anisotropy [27].

The electric charge may also be viewed as an extra degree of freedom in the general relativistic or extended gravity framework in the context of the construction of physically realistic models of compact stars. The introduction of a charge distribution makes it possible to discretely flex the FEs to produce precise or numerical solutions whilst preserving favorable physical properties like regularity at the centre, positivity of the density of energy, and causality of sound speeds [28]. The charge is added to the effective pressure and energy density by the electromagnetic field tensor, which alters the Tolman Oppenheimer Volkowoff (TOV) equation and affects the star equilibrium configuration. Thus by adding charge to the solution-generating process one can not only generate a wider range of compact star models, but also has a systematic means to investigate the effects of electromagnetism on stability, anisotropy, and observable properties such as mass-radius relations [29–31]. The inclusion of electric charge may also have important astrophysical consequences. Several studies indicate that intense gravitational collapse, phase transitions, or charge separation mechanisms in ultra-dense matter may support non-negligible local charge distributions. The resulting electromagnetic repulsion can partially counterbalance gravitational attraction, modify hydrostatic equilibrium, and permit more massive stable configurations. In this sense, charged solutions serve as useful theoretical probes of the interplay between gravitation and electromagnetism in extreme environments [32].

Many scientists have explored an embedding space-time metric for the formation of compact stars which fulfill Karmarkar condition. Karmarkar condition is a differential equation which appears while using embedding method by linking up relation between metric potentials [33]. For perfect fluid which is non static Gupta et al. [34] have considered plane symmetric metric to investigate the embedding class-I solutions. In [35], the authors developed a generalized and accurate framework for modeling compact anisotropic stars. To examine validity of generic solutions one may use different physical parameters like mass-radius relationship, compactness, redshift, energy conditions and stability. In the context of development of astrophysical framework Karmarkar condition method was used by Ospino et al. [36].

The most commonly used linear equation of state is a first-order approximation to the fundamental dependence of pressure and density between dark matter and in dense matter, but is not analytically tractable, and is often chosen to model compact stars [37,38]. The reason to adopt conformal flatness is that it can be used to reduce the Weyl tenor to zero, eliminating tidal forces and making it easier to interpret matter distribution in highly compact configurations. The Karmarkar condition gives a geometry embedding constraint, which brings the system of field equations to a single generating function, simplifying the solution procedure significantly but still giving realistic stellar models. Geometric restrictions (conformally flat and Karmarkar conditions) that are imposed between metric functions make it significantly easier to find solutions to the field equations, and provide well-behaved interior solutions to anisotropic compact stars [39].

The conformal symmetry of space-time are associated to the general framework. When a spacetime admits conformal symmetry, it necessarily possesses a conformal Killing vector field [40]. For the development of persistent system of equations, assumption of conformal flatness is more appropriate in most of the cases. In [41], the author examined the general form of conformal Killing vectors in spherically symmetric spacetimes.The reason behind conformal flatness adoption is that it can be used to reduce the Weyl tenor to zero, eliminating tidal forces and making it easier to interpret matter distribution in highly compact configurations. The Karmarkar condition gives a geometry embedding constraint, which brings the system of field equations to a single generating function, simplifying the solution procedure significantly but still giving realistic stellar models. Geometric restrictions (conformally flat and Karmarkar conditions) that are imposed between metric functions make it significantly easier to find solutions to the field equations, and provide well-behaved interior solutions to anisotropic compact stars Masan et al. [42] made comprehensive analysis about the motion (Kinematics and dynamics) of conformal killing vectors.

In a gravitating system the irregular pressure distribution is measured in term of anisotropy describing unequal pressure stresses. For constructing static, anisotropic, spherically symmetric solutions, Núñez et al. [43] developed a general technique that realizes a non-local EoS consistent with the prescribed density profile, under the condition of a vanishing Weyl tensor. Herrera et al. constructed Conformally flat interior solutions for locally anisotropic fluid to the Einstein equations [44]. Extending their findings to higher-dimensional spacetimes, Harko et al. [45] studied the hydrostatic equilibrium in a static anisotropic relativistic fluid sphere with a cosmological constant. Similarly, a number of classes of exact solutions to the Einstein FEs representing static, spherically symmetric anisotropic compact objects were given by Mak et al. [46]. Pressure anisotropy is a realistic ingredient in compact-star interiors. At extreme densities, anisotropic stresses may arise due to strong interactions, magnetic fields, viscosity, or multi-fluid composition. When radial and tangential pressures differ, the equilibrium and stability of the star are substantially affected. Positive anisotropy may support higher compactness, whereas negative anisotropy may enhance collapse tendencies. Hence, anisotropic models are often more realistic than ideal perfect-fluid approximations for dense stellar matter [47].

When the transverse pressure link up dominating energy criteria Ivanov calculated surface redshift of anisotropic compact objects [48]. The author in [49] discussed about relativistic fluids in the context of importance of anisotropy for spherically symmetric fluid. Many authors worked on models of compact objects [50–53]. In [54], the author presented solutions to the Einstein-Maxwell equations that address the problem of charged spheres within a one-parameter group of conformal motions, obtaining five analytical solutions for a static and spherically symmetric charge matter distribution. Thirukkanesh and Maharaj [55] studied anisotropic, relativistic, charged spheres that are related to compact stars. In the context of charged Krori–Barua spacetime solutions, Rahaman et al. [56] investigated ultra-compact astrophysical objects. Physically plausible models of charged compact stars were also covered by Kalam et al. [57].

In many fields of physics and mathematics, nonlinear ordinary differential equation of first order is known as the RE generally used. It is in the form


$$\begin{array}{c} \frac{dy}{dx}=J(x)y^{2}+K(x)y+L(x), \end{array}$$ (2)

here *J*(*x*), *K*(*x*) and *L*(*x*) are continuous functions of *x*. The general RE has many specific cases for distinct values of *J*(*x*), *K*(*x*) and *L*(*x*). Existence of an EoS describe a relation between pressure and density. The RE describes the relation between radial and transverse pressures [26]. For a solution of general RE, if a particular solution is known, then it can be constructed. There is no specific algorithm to find a certain solution consist on such functions [58–60].

Actually generating solution is a technique to illustrate from any already described solution to FEs containing killing vector, how one-parameter set of new solutions can be found. Transformations in already contained potentials are either continuous or discrete to form a new set of solutions [61,62].

The current work consists of the development of a general framework for charged spherically symmetric generating solutions in a modified theory for embedding class solutions. The arrangement of the paper is as follows: In sec. 2, we have developed FEs for a charged spherically symmetric compact object and anisotropy factor. In sec. 3, we focused on finding generating solutions with the help of the Karmarkar condition and conformal motion. In sec. 4, we present a detailed discussion of our results.

## 2 Development of modified field equations

We consider a static spherically symmetric line element for a compact object to be given as


$$\begin{array}{c} ds^{2}=e^{\mu (r)}dt^{2}-e^{\nu (r)}dr^{2}-r^{2}(d\theta^{2}+\sin^{2}\theta d\phi^{2}), \end{array}$$ (3)

where $e^{\mu (r)}$ and $e^{\nu (r)}$ represent metric potentials/coefficients, and modified FEs are given by


$$\begin{array}{c} G_{\alpha \beta }=R_{\alpha \beta }-\frac{1}{2}Rg_{\alpha \beta }=\frac{\kappa }{f_{R}}\left[T_{\alpha \beta }^{\left(m\right)}+T_{\alpha \beta }^{\left(eff\right)}+E_{\alpha \beta }\right]. \end{array}$$ (4)

In FEs, the Einstein tensor $G_{\alpha \beta }$ represents the geometric part and corresponds to the metric (3). On right hand, $T_{\alpha \beta }^{\left(m\right)}$ contributes usual matter energy momentum tensor and $E_{\alpha \beta }$ is electromagnetic tensor. The contribution of dark components can be determined through the effective energy momentum tensor $T_{\alpha \beta }^{\left(eff\right)}$. The usual matter distribution for anisotropic pressure stresses given by


$$\begin{array}{c} T_{\beta }^{\alpha }{\;}^{\left(m\right)}=(\rho +p_{r})U^{\alpha }U_{\beta }+p_{t}g_{\beta }^{\alpha }+(p_{r}-p_{t})V^{\alpha }V_{\beta }, \end{array}$$ (5)

where $p_{r}$ and $p_{t}$ represents radial and transverse pressures and ρ is the energy density, $U^{\alpha }$ denote 4-velocity and $V^{\alpha }$ stands for radial 4-vector. These quantities satisfy the conditions $U^{ɩ}U_{ȷ}=-V^{ɩ}V_{ȷ}=1$ and $U^{ɩ}V_{ȷ}=0$.

With the help of the metric variational principle, taking variation in (1) leads to the following form [3]:


$$\begin{array}{c} R_{\alpha \beta }f_{R}-\frac{1}{2}f(R)g_{\alpha \beta }+(g_{\alpha \beta }◻-\nabla_{\alpha }\nabla_{\beta })f_{R}=\kappa T_{\alpha \beta }^{\left(m\right)}, \end{array}$$ (6)

where $f_{R}=\frac{df}{dR}$, $T_{\mu \nu }$ represents the matter energy momentum tensor, $◻\equiv \nabla^{\alpha }\nabla_{\alpha }$ denotes the de’Alembertian operator, and $\nabla_{\alpha }$ is the covariant derivative.

The effective energy-momentum tensor, which incorporates dark-source contributions, is defined as $T_{\alpha \beta }^{\left(eff\right)}$:


$$\begin{array}{c} T_{\alpha \beta }^{\left(eff\right)}=\frac{1}{\kappa }\left[\frac{f-Rf_{R}}{2}g_{\alpha \beta }+(\nabla_{\alpha }\nabla_{\beta }-g_{\alpha \beta }◻)f_{R}\right], \end{array}$$ (7)

Ricci scalar is given by


$$\begin{array}{c} R=\frac{-\mu^{\prime \prime }}{e^{\nu }}-\frac{(\mu^{\prime }{)}^{2}}{2e^{\nu }}+\frac{\mu^{\prime }\nu^{\prime }}{2e^{\nu }}+\frac{2\mu^{\prime }}{re^{\nu }}-\frac{2\nu^{\prime }}{re^{\nu }}-\frac{2}{r^{2}e^{\nu }}+\frac{2}{r^{2}}. \end{array}$$ (8)

$E_{\mu \nu }$ represents electromagnetic energy-momentum tensor and is given as,


$$\begin{array}{c} E_{\mu \nu }=\frac{1}{4\pi }\left(F_{\mu }^{\alpha }F_{\alpha \nu }-\frac{1}{4}F^{\alpha \beta }F_{\alpha \beta }g_{\mu \nu }\right), \end{array}$$ (9)

Now $E_{\mu \nu }$ represents the electromagnetic field strength tensor that satisfies the Maxwell equations.


$$\begin{array}{c} F_{\mu \nu };\lambda +F_{\nu \lambda };\mu +F_{\mu \lambda };\nu =0, \end{array}$$ (10)

The non-zero components of the electromagnetic field tensor are *F*^01^ and *F*^10^


$$\begin{array}{c} F^{01}=-F^{10}, \end{array}$$

From Eq. (10) the electric field component can be written as.


$$\begin{array}{c} F^{01}=e^{\frac{\lambda +\nu }{2}}\frac{q(r)}{r^{2}}. \end{array}$$ (11)

The set of modified FEs is given by


$$\begin{array}{c} G_{00}=\frac{e^{\mu }}{f_{R}}\left[\frac{f-Rf_{R}}{2}+e^{-\nu }(f_{R}^{\prime \prime }+\frac{2}{r}f_{R}^{\prime }-\frac{\nu^{\prime }}{2}f_{R}^{\prime })\right]+\frac{8\pi }{f_{R}}(\rho +\frac{E^{2}}{8\pi })e^{\mu }, \end{array}$$ (12)

$$\begin{array}{c} G_{11}=\frac{1}{f_{R}}\left[-\frac{f-Rf_{R}}{2}e^{\nu }-\frac{2}{r}f_{R}^{\prime }-\frac{\mu^{\prime }}{2}f_{R}^{\prime }\right]+\frac{8\pi }{f_{R}}(p_{r}-\frac{E^{2}}{8\pi })e^{\nu }, \end{array}$$ (13)

$$\begin{array}{c} \begin{array}{c} G_{22}=\frac{1}{f_{R}}\left[-\frac{f-Rf_{R}}{2}r^{2}-e^{-\nu }(rf_{R}^{\prime }-r^{2}f_{R}^{\prime \prime }-\frac{r^{2}\mu^{\prime }}{2}f_{R}^{\prime }+\frac{r^{2}\nu^{\prime }}{2}f_{R}^{\prime })\right]\\ \;+\frac{8\pi }{f_{R}}(p_{t}+\frac{E^{2}}{8\pi })r^{2}, \end{array} \end{array}$$ (14)

$$\begin{array}{c} \begin{array}{c} G_{33}=\frac{1}{f_{R}}[-\frac{f-Rf_{R}}{2}r^{2}\sin^{2}\theta -e^{-\nu }(r\sin^{2}\theta f_{R}^{\prime }-r^{2}\sin^{2}\theta f_{R}^{\prime \prime }-\frac{r^{2}\sin^{2}\theta \mu^{\prime }}{2}f_{R}^{\prime }\\ \;+\frac{r^{2}\sin^{2}\theta \nu^{\prime }}{2}f_{R}^{\prime })]+\frac{8\pi }{f_{R}}(p_{t}+\frac{E^{2}}{8\pi })r^{2}\sin^{2}\theta . \end{array} \end{array}$$ (15)

Here (′) denotes the differentiation with respect to ’*r*’. After simplification (12)-(15), taking κ=8π we get


$$\begin{array}{c} \begin{array}{c} \rho =\frac{e^{-\nu }}{16\pi r^{2}}[r^{2}\nu^{\prime }f_{R}^{\prime }-r^{2}e^{\nu }f-2r\nu^{\prime }f_{R}+r^{2}e^{\nu }Rf_{R}-2e^{\nu }f_{R}-2r^{2}f_{R}^{\prime \prime }-4rf_{R}^{\prime }+2f_{R}]\\ \;-\frac{E^{2}}{8\pi }, \end{array} \end{array}$$ (16)

$$\begin{array}{c} \begin{array}{c} p_{r}=-\frac{e^{-\nu }}{16\pi r^{2}}[2r\mu^{\prime }f_{R}+2f_{R}-2e^{\nu }f_{R}-e^{\nu }r^{2}f+e^{\nu }r^{2}Rf_{R}-4rf_{R}^{\prime }-r^{2}\mu^{\prime }f_{R}^{\prime }]\\ \;+\frac{E^{2}}{8\pi }, \end{array} \end{array}$$ (17)

$$\begin{array}{c} \begin{array}{c} p_{t}=\frac{e^{-\nu }}{32\pi r}[2\mu^{\prime }f_{R}-2\nu^{\prime }f_{R}+2r\mu^{\prime \prime }f_{R}+r(\mu^{\prime }{)}^{2}f_{R}-r\mu^{\prime }\nu^{\prime }f_{R}-2re^{\nu }f+2re^{\nu }Rf_{R}\\ \;-4(f_{R}^{\prime }+rf_{R}^{\prime \prime })-2r(\mu^{\prime }f_{R}^{\prime }-\nu^{\prime }f_{R}^{\prime })]-\frac{E^{2}}{8\pi }. \end{array} \end{array}$$ (18)

The Eqs. (16)–(18) describes a set of modified FEs consist only first and second order derivatives of μ, to associate it with the four-acceleration *a*_1_, with transformation 2*a*_1_ = $\mu^{\prime }$ [26]. The system consists of three equations with five unknowns such as ρ, $p_{r}$, $p_{t}$, μ and ν. We can take randomly two of them, where under consideration model is physically viable if it satisfy a number of conditions like regularity, stability and matching conditions too. Distinct constraints can be applied on the modified FEs. The EoS $p_{r}$ = χ(ρ) is the existence of constraint. It is important to describe the anisotropy measure between the matter configuration denoted by Δ = $p_{t}$ – $p_{r}$ which is called anisotropy factor. For this using Eqs. (17) and (18) we have


$$\begin{array}{c} \Delta =\frac{e^{-\nu }}{8\pi r}[\frac{r\mu^{\prime \prime }f_{R}}{2}-\frac{r\mu^{\prime }\nu^{\prime }f_{R}}{4}+\frac{r(\mu^{\prime }{)}^{2}f_{R}}{4}+\frac{r\nu^{\prime }f_{R}^{\prime }}{2}-rf_{R}^{\prime \prime }+\frac{f_{R}}{r}\\ -(\frac{\nu^{\prime }}{2}-\frac{e^{\nu }}{r})f_{R}-(r\mu^{\prime }+\frac{3\mu^{\prime }}{2}-3)f_{R}^{\prime }-re^{\nu }f+re^{\nu }Rf_{R}]-\frac{2E^{2}}{8\pi }. \end{array}$$ (19)

We apply two conditions on the space-time to get linear RE. We apply conformally flat condition first, which occurs when the value of Weyl tensor vanishes [63–66]. If Killing vectors *K* occur, it is a particular and specific case of space-times with conformal motion satisfying the following equation.


$$\begin{array}{c} L_{K}g_{ab}=2\psi g_{ab}, \end{array}$$ (20)

Where $L_{K}$ and ψ(t,r) represent the Lie derivative algorithm and conformal component, respectively. [26] describes the following equation as


$$\begin{array}{c} 2\mu^{\prime \prime }r^{2}+(\mu^{\prime }{)}^{2}r^{2}=\nu^{\prime }\mu^{\prime }r^{2}+2r\mu^{\prime }-2r\nu^{\prime }+4e^{\nu }(1+\zeta )-4, \end{array}$$ (21)

where “ζ” is a integration constant and for conformally flat spacetime if ζ = 0. we left


$$\begin{array}{c} 2\mu^{\prime \prime }r^{2}+(\mu^{\prime }{)}^{2}r^{2}=\nu^{\prime }\mu^{\prime }r^{2}+2r\mu^{\prime }-2r\nu^{\prime }+4e^{\nu }-4, \end{array}$$ (22)

$$\begin{array}{c} 2\mu^{\prime \prime }+(\mu^{\prime }{)}^{2}=\nu^{\prime }\mu^{\prime }+\frac{2}{r}(\mu^{\prime }-\nu^{\prime })+\frac{4}{r^{2}}(e^{\nu }-1). \end{array}$$ (23)

Recently, many researchers have worked on the class one embedding of space-times. By using the Karmarkar condition, we apply a technique in order to obtain static spherical solutions for embedding class one. The Karmarkar condition links up two main parts of the metric potentials of a spherically symmetric space-time. They may be incorporated within a flat space-time. For accomplish this the components of Riemann’s tensor and Karmarkar connection must be coupled [33].


$$\begin{array}{c} R_{hijk}=\pm (g_{hj}g_{ik}-g_{ij}g_{hk}), \end{array}$$ (24)

After eliminating the zero component of above equation, we get


$$\begin{array}{c} R_{1010}R_{2323}-R_{1212}R_{3030}=R_{1220}R_{1330}, \end{array}$$ (25)

that leads to the following differential equation


$$\begin{array}{c} \frac{2\mu^{\prime \prime }}{\mu^{\prime }}+\mu^{\prime }=\frac{\nu^{\prime }e^{\nu }}{e^{\nu }-1}. \end{array}$$ (26)

Equations (16)–(19) and (21) exhibit linearity in the variable *y* = $e^{-\nu }$, while Eq. (26) is linear in *y* = $e^{\nu }$. It is important to note that Eq. (16) does not contain parameter *a*_1_, whereas Eq. (17) explicitly contains *a*_1_. The remaining equations take the form of either Bernoulli or Riccati differential equations with respect to *a*_1_. To simplify the analysis, these nonlinear equations can be transformed into linear form by introducing the substitution $u=e^{\frac{\mu }{2}}$. This transformation is particularly advantageous, as it converts the nonlinear Riccati equation into a second-order linear ordinary differential equation. Consequently, standard linear solution techniques and well-established classes of solutions can be effectively applied [59]. The RE is given by


$$\begin{array}{c} g\frac{dy}{dx}=f_{2}y^{2}+f_{1}y+f_{0}, \end{array}$$ (27)

where *f*_0_, *f*_1_, *f*_2_, and *g* are functions of the independent variable *r*. To illustrate special cases, we first consider the linear and Bernoulli forms of Eq. (27). The linear case is obtained when *f*_2_ = 0, reducing Eq. (27) to


$$\begin{array}{c} g\frac{dy}{dx}=f_{1}y+f_{0} \end{array}$$ (28)

Eq. (28) is directly integrable and its solution can be expressed as


$$\begin{array}{c} y=(C+\int exp(-G)\frac{f_{0}}{g}dr)exp(G),\;G=\int \frac{f_{1}}{g}dr \end{array}$$ (29)

Next, we consider the Bernoulli equation, obtained when *f*_0_ = 0 and n≠1:


$$\begin{array}{c} g\frac{dy}{dx}=f_{1}y+f_{n}y^{n},\;y^{-n}gy^{\prime }=f_{1}y^{1-n}+f_{n}, \end{array}$$ (30)

So its solution is


$$\begin{array}{c} y^{1-n}=Cexp(G)+(1-n)exp(G)\int exp(-G)\frac{f_{n}}{g}dr,\;G=(1-n)\int \frac{f_{1}}{g}dr. \end{array}$$ (31)

$$\begin{array}{c} g\frac{dy}{dr}=f_{1}y+f_{n}y^{n}. \end{array}$$ (32)

Dividing by $y^{n}$, Eq. (32) can be rewritten as


$$\begin{array}{c} y^{-n}g\frac{dy}{dr}=f_{1}y^{1-n}+f_{n}. \end{array}$$ (33)

Its general solution is then given by


$$\begin{array}{c} y^{1-n}=Ce^{G}+(1-n)e^{G}\int e^{-G}\frac{f_{n}}{g}\;dr,\;G=(1-n)\int \frac{f_{1}}{g}\;dr. \end{array}$$ (34)

It is well known that the Riccati equation given in Eq. (27) does not admit a general closed-form solution. However, certain special cases are integrable. For instance, when *f*_2_ = 0, Eq. (27) reduces to a linear differential equation, which can be solved exactly [33]. On the other hand, when *f*_0_ = 0 and *n* = 2, Eq. (27) takes the form of a Bernoulli equation, which also admits a general solution. In this case, the solution can be written as


$$\begin{array}{c} y=Ce^{G}+e^{G}\int e^{-G}\frac{f_{0}}{g}dr,\;G=\int \frac{f_{1}}{g}dr. \end{array}$$ (35)

Moreover, by introducing the transformation $z=\frac{1}{y}$, the Riccati equation can be converted into an integrable linear equation in *z*. Furthermore, the Riccati equation can be transformed into a second-order homogeneous linear differential equation by introducing a new function *u* [35]. In the particular case when $f_{2}=-g$, the transformed equation takes the form


$$\begin{array}{c} f_{2}u^{\prime \prime }+f_{1}u^{\prime }+f_{0}u=0,\;u=exp\int ydr. \end{array}$$ (36)

Substituting $y=\frac{u^{\prime }}{u}$ into Eq. (27) recovers the original Riccati equation, confirming the consistency of the transformation. It is important to note that the parameter *a*_1_ does not appear in the expression for the energy density; therefore, it cannot be employed as a generating function.


$$\begin{array}{c} y=e^{-\nu }, \end{array}$$ (37)

Eq. (16) can be rewritten in the form of *y* as


$$\begin{array}{c} \frac{y^{\prime }}{16\pi r^{2}}(-r^{2}f_{R}^{\prime }+2rf_{R})=\rho -\frac{1}{8\pi }[\frac{y}{r^{2}}(f_{R}-r^{2}f_{R}^{\prime \prime }-2rf_{R}^{\prime })+\frac{f-Rf_{R}}{2}+\frac{f_{R}}{r^{2}}+E^{2}]. \end{array}$$ (38)

The expression of the energy density above offers a platform on which new classes of generating solutions can be built in the context of modified *f*(*R*) gravity.

## 3 Generating solutions

In the following subsections, we derive generating solutions within the framework of modified gravity by solving Eqs. (17)–(19).

### 3.1 Derivation of solutions from the radial pressure condition

From Eq. (17) we derive the generating solution for radial pressure $p_{r}$. Making use of Eq. (37) into Eq. (17) along with metric potentials given in Eq. (3), We obtain the following form


$$\begin{array}{c} 16\pi r^{2}p_{r}=(4rf_{R}^{\prime }-2f_{R}-4a_{1}rf_{R}+2a_{1}r^{2}f_{R}^{\prime })y+2f_{R}-r^{2}Rf_{R}+r^{2}f+2r^{2}E^{2}. \end{array}$$ (39)

leading to


$$\begin{array}{c} \mu^{\prime }=2a_{1}=\frac{16\pi r^{2}p_{r}-2f_{R}+r^{2}Rf_{R}-r^{2}f-2r^{2}E^{2}-y(4rf_{R}^{\prime }-2f_{R})}{ry(rf_{R}^{\prime }-2f_{R})}. \end{array}$$ (40)

A simple generating function involving *y*, *a*_1_ and $p_{r}$ can be obtain from Eq. (39). For related approaches and solutions involving specific forms of $p_{r}$ and *y*, the reader is referred to Refs. [67–69]. Now, by adopting ageneral EoS of the form $p_{r}=\chi (\rho )$ and substituting Eq. (38) into Eq. (39), we obtain


$$\begin{array}{ccc} & & 2a_{1}y(r^{2}f_{R}^{\prime }-2rf_{R})=-2f_{R}+Rf_{R}r^{2}-r^{2}f-(-2f_{R}+4rf_{R}^{\prime })y+[y^{\prime }(-r^{2}f_{R}^{\prime }\\ & & +2rf_{R})+2y(f_{R}-r^{2}f_{R}^{\prime \prime }-2rf_{R}^{\prime })-r^{2}f+r^{2}Rf_{R}-2f_{R}-4r^{2}E^{2}]. \end{array}$$ (41)

Eq. (41) represents a nonlinear relationship between the metric potentials and the parameter *a*_1_ in terms of *y*. To proceed further, we consider a special case of the general EoS, namely the linear EoS given by


$$\begin{array}{c} p_{r}=a\rho -b, \end{array}$$ (42)

where b≥0 is the bag constant and *a* is a constant satisfying 0≤a≤1. Linear EoS has good reasons to be adopted in the modeling of charged compact objects, especially in quark stars and bag models. In these systems, matter is thought to have been deconfined in a quark state of matter, where the pressure is a linear relationship to the energy density.This linear relation has been useful in explaining the physical behavior of ultra-dense matter at high energies. It is also worth noting that the case $p_{r}=0$ can be recovered as a particular instance of this EoS [70]. Consequently, Eq. (40) reduces to


$$\begin{array}{c} \begin{array}{c} a(-r^{2}f_{R}^{\prime }+2rf_{R})y^{\prime }=-y(4a_{1}rf_{R}-2a_{1}r^{2}f_{R}-4rf_{R}^{\prime }+2f_{R}+2af_{R}\\ \;+2ar^{2}f_{R}^{\prime \prime }+4raf_{R}^{\prime })-f_{R}(-2+ar^{2}R-2a+r^{2}R)+r^{2}f(a+1)+16\pi br^{2}\\ \;+2r^{2}E^{2}(a+1). \end{array} \end{array}$$ (43)

The linear RE in terms of *y* is obtained from Eq. (43), and the unknowns *a*, *a*_1_, and *b* can be solved for using Eq. (35). For the solution of above DE Eq. (43) integrating factor calculated as H=∫Ω(r)dr, where


$$\begin{array}{c} \Omega (r)=\frac{4a_{1}rf_{R}-2a_{1}r^{2}f_{R}-4rf_{R}^{\prime }+2f_{R}+2af_{R}+2ar^{2}f_{R}^{\prime \prime }+4raf_{R}^{\prime }}{a(2rf_{R}-r^{2}f_{R}^{\prime })}. \end{array}$$ (44)

After simplification as mentioned above we get the generic solution of linear Riccati Eq. (43) becomes


$$\begin{array}{c} y=e^{-G}\int \frac{(-f_{R}(-2+ar^{2}R-2a+r^{2}R)+r^{2}f(a+1)+16\pi br^{2}+2r^{2}E^{2}(a+1))e^{G}dr}{a(2rf_{R}-r^{2}f_{R}^{\prime })}+C_{1}, \end{array}$$ (45)

where *C*_1_ is a constant of integration and can be calculated using the initial condition at *r* = 0, $e^{G}=0$, giving *C*_1_ = 0.

### 3.2 Derivation of solutions from the transverse pressure condition

From Eq. (18) of transverse pressure $p_{t}$ a linear RE can be derived for *y* as mentioned above


$$\begin{array}{c} \begin{array}{cc} & y^{\prime }(-\frac{a_{1}}{16\pi }f_{R}+\frac{1}{16\pi }f_{R}^{\prime }-\frac{1}{16\pi r}f_{R})=y(\frac{a_{1}}{8\pi r}f_{R}+\frac{a_{1}^{2}}{8\pi }f_{R}+\frac{a_{1}^{\prime }}{8\pi }f_{R}-\frac{1}{8\pi r}f_{R}^{\prime }\\ & +\frac{a_{1}}{8\pi }f_{R}^{\prime }-\frac{1}{8\pi }f_{R}^{\prime \prime })+\frac{1}{16\pi }Rf_{R}-\frac{f}{16\pi }-\frac{E^{2}}{8\pi }-p_{t}. \end{array} \end{array}$$ (46)

The solution of above linear Riccati Eq. (46) using Eq. (35) can be rewritten as


$$\begin{array}{c} y=[C_{2}+\int e^{F}(\frac{rRf_{R}-rf-2rE^{2}-16\pi rp_{t}}{-a_{1}rf_{R}+rf_{R}^{\prime }-f_{R}})dr]e^{-F}, \end{array}$$ (47)

where


$$\begin{array}{c} e^{F}=e^{\int 2\delta (r)dr}, \end{array}$$ (48)

and


$$\begin{array}{c} \delta (r)=\frac{a_{1}^{\prime }rf_{R}+a_{1}^{2}rf_{R}+a_{1}rf_{R}^{\prime }+a_{1}f_{R}-rf_{R}^{\prime \prime }-f_{R}^{\prime }}{-f_{R}-a_{1}rf_{R}+rf_{R}^{\prime }}. \end{array}$$ (49)

Applying the initial condition *r* = 0 with $e^{F}=0$ yields *C*_2_ = 0. Substituting this into Eq. (46), a linear RE for *a*_1_ can be obtained as


$$\begin{array}{ccc} \frac{a_{1}^{\prime }}{8\pi }yf_{R} & = & \frac{a_{1}^{2}}{8\pi }yf_{R}+(\frac{y}{8\pi r}f_{R}+\frac{y}{8\pi }f_{R}^{\prime }-\frac{y^{\prime }}{16\pi }f_{R})a_{1}+(\frac{1}{16\pi }f_{R}^{\prime }-\frac{1}{16\pi r}f_{R})y^{\prime }\\ & & -(\frac{1}{8\pi }f_{R}^{\prime \prime }+\frac{1}{8\pi r}f_{R}^{\prime })y+\frac{1}{16\pi }f-\frac{R}{16\pi }f_{R}+p_{t}+\frac{E^{2}}{8\pi }, \end{array}$$ (50)

simplification of Eq. (50) for known values of *y* and $p_{t}$. Moreover it can be transformed into a linear homogeneous DE of order two following Eqs. (35) and (36) as


$$\begin{array}{c} \frac{yf_{R}}{8\pi }u^{\prime \prime }-(\frac{y}{8\pi r}f_{R}+\frac{y}{8\pi }f_{R}^{\prime }-\frac{y^{\prime }}{16\pi }f_{R})u^{\prime }-[(\frac{f_{R}^{\prime }}{16\pi }-\frac{f_{R}}{16\pi r})y^{\prime }\\ -(\frac{f_{R}^{\prime }}{8\pi r}+\frac{f_{R}^{\prime \prime }}{8\pi })y+(\frac{f}{16\pi }-\frac{Rf_{R}}{16\pi }+p_{t})+\frac{E^{2}}{8\pi }]u=0, \end{array}$$ (51)

where


$$\begin{array}{c} u=e^{\frac{\mu }{2}}. \end{array}$$ (52)

In certain cases, Eq. (51) can be obtained more easily than the original Riccati equation, while still allowing the discussion of many important functions. By assuming $y=e^{-\nu }$, we reduce the problem to a first-order linear differential equation, which is directly integrable. This approach can be regarded as a “double linear” formulation. Consequently, if any two of the quantities $p_{t}$, *y*, and *a*_1_ are specified, the remaining variable, $p_{t}$, can serve as a generating function for constructing solutions in modified gravity models of stellar objects.

### 3.3 Generating solution from anisotropy factor

Following the same procedure which we have already discussed about calculation of generating solution. we will find the generating solution using anisotropy factor Δ given in Eq. (19).


$$\begin{array}{c} y^{\prime }(\frac{rf_{R}}{2}(a_{1}+\frac{1}{r})-\frac{r}{2}f_{R}^{\prime })=-rf_{R}(a_{1}^{\prime }+a_{1}^{2}+\frac{1}{r^{2}})y+ryf_{R}^{\prime \prime }+\\ f_{R}^{\prime }(3+2a_{1}r-3a_{1})y+(rf+\frac{f_{R}}{r}-rRf_{R}-8\pi r\Delta +2rE^{2}), \end{array}$$ (53)

Equation (53) is a linear RE in term of *y*.

By putting $z=a_{1}+\frac{1}{r}$, Eq. (53) becomes


$$\begin{array}{c} y^{\prime }=\frac{1}{\left(\frac{rf_{R}}{2}(z)-\frac{r}{2}f_{R}^{\prime }\right)}[-rf_{R}((z^{\prime }+z^{2}-\frac{2a_{1}}{r}+\frac{1}{r^{2}})+rf_{R}^{\prime \prime }+f_{R}^{\prime }\\ (3+2a_{1}r-3a_{1}))y+(rf-rRf_{R}+\frac{f_{R}}{r}+2rE^{2}-8\pi r\Delta )], \end{array}$$ (54)

the solution of above equation can be written as [71]


$$\begin{array}{c} y=e^{-G}C_{3}-e^{-G}\int e^{G}\frac{\left(-rRf_{R}+rf+\frac{f_{R}}{r}+2rE^{2}-8\pi r\Delta \right)}{\left(\frac{rf_{R}}{2}(z)-\frac{r}{2}f_{R}^{\prime }\right)}dr, \end{array}$$ (55)

where


$$\begin{array}{c} e^{G}=e^{\int \omega (r)dr}, \end{array}$$ (56)

and


$$\begin{array}{c} \omega (r)=\frac{\left(-rf_{R}(z^{\prime }+z^{2}-\frac{2a_{1}}{r}+\frac{1}{r^{2}})y+rf_{R}^{\prime \prime }y+f_{R}^{\prime }(2a_{1}r-3a_{1}+3\right)}{\left(\frac{rf_{R}}{2}(z)-\frac{r}{2}f_{R}^{\prime }\right)}. \end{array}$$ (57)

Eq. (53), can be rewrite as a linear RE for *a*_1_.


$$\begin{array}{cc} 2a_{1}^{\prime }y= & -2a_{1}^{2}y+a_{1}(-y^{\prime }-\frac{6yf_{R}^{\prime }}{rf_{R}}+\frac{4yf_{R}^{\prime }}{f_{R}})+(\frac{ry^{\prime }-2y+6ry\frac{f_{R}^{\prime }}{f_{R}}+2}{r^{2}})\\ & +\frac{f_{R}^{\prime }}{f_{R}}y^{\prime }+\frac{1}{f_{R}}(2yf_{R}^{\prime \prime }+2f-16\pi \Delta +4E^{2})-2R, \end{array}$$ (58)

Following Eq. (36) it may be transformed into linear form as.


$$\begin{array}{cc} & 2yu^{\prime \prime }-(y^{\prime }-\frac{6yf_{R}^{\prime }}{rf_{R}}+\frac{4yf_{R}^{\prime }}{f_{R}})u^{\prime }-((\frac{ry^{\prime }-2y+6ry\frac{f_{R}^{\prime }}{f_{R}}+2}{r^{2}})-\frac{f_{R}^{\prime }}{f_{R}}y^{\prime }\\ & +\frac{1}{f_{R}}(2yf_{R}^{\prime \prime }+2f-16\pi \Delta +4E^{2})-2R)u=0. \end{array}$$ (59)

So, Eq. (59) is double linear, same as Eq. (51). If any two constants Δ, *y* and *a*_1_ are known, Eq. (19) can be used as a generating function in *f*(*R*) as a solution for stellar models.

## 4 Conclusion

Extended theories of gravity have grabbed the attention of many researchers due to combined motivation in the field of high energy physics, cosmology and astrophysics. Modified theories of gravity has revealed so many hidden facts about our universe and shown that it has the same bases. One of the most well known theory of gravity is *f*(*R*) gravity. we have focused on spherically symmetric charged compact object in *f*(*R*) gravity and developed basic formulation of FEs Eqs. (12)-(15) using Einstein Maxwell charged component.

The modified Einstein-Maxwell equations are used to describe the combined interaction of gravity and electromagnetism when two important effects, which are gravitational and electromagnetic effects are used. These equations describe both Einstein’s field equations for gravity and Maxwell’s equations for electromagnetism into a single framework. As Einstein’s theory is strongly based on equivalence principle which sets the stage for how charge interact with gravity. In classical electromagnetism as described by Maxwell’s equations, electric charges create electric fields and it incorporates how gravity effects on charge. Theory of GR predicts that the presence of mass and energy affects electromagnetic fields as well. Therefore, It is vital to study impact of charge in modified gravities and more specifically generating solutions for this setup.

In this article, we have developed generating functions which provide solutions to modified FEs constrained by conformal symmetry and EoS for compact objects. The modified FEs, given by Eqs. (16)–(18), are nonlinear. To obtain solutions of embedding class one, we employ the Karmarkar condition together with the conformal flatness constraint. The conformal Killing vectors have a significant contribution in adding a symmetry that makes the field equations simpler and allows them to be used to construct exact solutions. The conformal symmetry brought about by these vectors simplifies the nonlinear field equations and maintains the underlying geometry of spacetime structure [72,73].

For this three distinct cases have been taken under consideration to obtain generating solutions in *f*(*R*) gravity. We have shown that modified FEs (17)-(19) for two pressures $p_{r}$, $p_{t}$ and the anisotropy factor Δ = $p_{t}$ – $p_{r}$ may be used as generating functions. It can also be a generating function for perfect fluid models when Δ = 0. However, the first modifying FE (16) for ρ does not consist *a*_1_, so we cannot use it in the development of generating solution. When ρ term is used in EoS, then combination of Eqs. (16)-(17) can be used as a generating solution providing a relation between the two metric potentials. For development of linear RE in modified gravity, space-time encompassed by two conditions which are elimination of Karmarkar condition for class-I metric and Weyl tensor. Now we are going to discuss a detailed explanation of three cases.

- First and simplest generating solution for embedding class-I has been obtained using modified FE for radial pressure. It has been obtained using transformation from Eq. (37) and linear EoS Eq. (42). Eq. (43) present a linear RE in term of *y* and its solution has been obtained by showing integrating factor in Eq. (45). With the help of initial condition *r* = 0 for particular solution constant of integration has been calculated which is $e^{\left(F\right)}=0$.
- Now another generating solution which is calculated using third modified FE for transverse pressure. Following the same procedure the transformation given in Eq. (37) is used to obtain linear RE in term of *y* given by Eq. (46). It has been solved using integrating factor shown in Eq. (47) and its particular solution has been obtained using initial condition. Another type of linear RE is obtained in *a*_1_, which is then used to develop a double linear equation. In addition, employing Eq. (52) in Eq. (50) yields a linear homogeneous DE of second order in the term of *u*.
- Eq. (19) is used to obtain third generating solution for anisotropy factor Δ. For this we implement transformation of Eq. (37) on Eq. (19) and follow the same criteria to get generating solution in Eq. (55). A double linear RE has been obtained in *a*_1_. Also by using Eq. (52) in Eq. (58) a linear homogeneous DE of second order has been developed in Eq. (59) in term of *u*.

In this work development of new solutions with the help of a single parameter has been done. For this through transformation already mentioned potentials are transformed into new one. It is important to note that present paper is about general framework on generating solutions for charged objects and it is possible to obtain in the systems of *f*(*R*) gravity when some particular solution is known. It is also shown that anisotropy factor may serves as important and basic source of generating solutions.

The generating function must guarantee regularity at the center, and positive and finite energy density and pressure, monotonic reduction of the thermodynamic variables, energy and causality conditions, perturbation stability, and smooth matching of the generated solutions to an appropriate exterior spacetime at the boundary, so that the generated solutions should correspond to physically admissible stellar solutions [74,75]. Within this context, the present work develops a framework for generating solutions of charged compact objects, providing a systematic method to construct families of physically relevant stellar models in modified gravity. Once a physically acceptable generating function is chosen, the corresponding density, pressures, and metric potentials can be examined under standard viability criteria such as positivity of matter variables and monotonic decrease of density and pressure profiles. Therefore, the framework developed here has direct astrophysical utility for exploring realistic compact-star structures in alternative gravity theories.
